# Mental health priorities in Vietnam: a mixed-methods analysis

**DOI:** 10.1186/1472-6963-10-257

**Published:** 2010-09-02

**Authors:** Maria Niemi, Huong T Thanh, Tran Tuan, Torkel Falkenberg

**Affiliations:** 1 Unit for Studies of Integrative Health Care, Division of Nursing, Department of Neurobiology, Caring Sciences and Society, Karolinska Institutet, (Alfred Nobels Allée 23), Huddinge, (141 83), Sweden; 2 Department of Education, Hanoi Medical University, (1 Ton That Tung) Hanoi, (Dong Da), Vietnam; 3 Non-Communicable Disease Control Unit, Viet Nam Ministry of Health, (138A Giang Vo), Hanoi, (Ba Dinh), Vietnam; 4 Research and Training Centre for Community Development, (No. 39, lane 255, Vong street) Hanoi, (Hai Ba Trung), Vietnam; 5 Research Unit, Vidar Clinic Foundation, (Ytterjärna), Järna, (15391) Sweden

## Abstract

**Background:**

The Mental Health Country Profile is a tool that was generated by the International Mental Health Policy and Services Project to inform policy makers, professionals and other key stakeholders about important issues which need to be considered in mental health policy development. The Mental Health Country Profile contains four domains, which include the mental health context, resources, provision and outcomes. We have aimed to generate a Mental Health Country Profile for Vietnam, in order to highlight the strengths and weaknesses of the Vietnamese mental health situation, in order to inform future reform efforts and decision-making.

**Methods:**

This study used snowball sampling to identify informants for generating a Mental Health Country Profile for Vietnam, and the data gathering was done through semi-structured interviews and collection of relevant reports and documents. The material from the interviews and documents was analysed according to qualitative content analysis.

**Results:**

Marked strengths of the Vietnam mental health system are the aims to move toward community management and detection of mental illness, and the active involvement of several multilateral organizations and NGOs. However, there are a number of shortages still found, including the lack of treatment interventions apart from medications, the high proportion of treatments to be paid out-of-pocket, prominence of large tertiary psychiatric hospitals, and a lack of preventative measures or mental health information to the public.

**Conclusions:**

At the end of this decade, mental health care in Vietnam is still characterised by unclear policy and poor critical mass especially within the governmental sector. This initial attempt to map the mental health situation of Vietnam suffers from a number of limitations and should be seen as a first step towards a comprehensive profile.

## Background

Neuropsychiatric diseases are the most important cause of disability in adults over 15 years old, causing 37% of the years lived with disability [1]. Despite this, most low- and middle income countries allocate little finances, and have poor infrastructure and human resources for mental health care [2].

There is a lack of commitment to mental health issues in many countries. For example, 45% of countries in South-East Asia have no mental health policy and one third have no national mental health program or mental health legislation. Fifty percent of these countries have no mental health care in the community, 55% have no treatment of severe mental disorders in primary care, and large tertiary institutions are still the main means of care. Twenty six percent of countries in South-East Asia do not have essential psychotropic drugs available in primary care [3].

Formative evaluation of policy can assist those responsible for a programme to shape it while it is being designed or implemented [4]. The Mental Health Country Profile is a tool for formative evaluation of the mental health situation in a country, and was generated by the International Mental Health Policy and Services Project to inform key stakeholders about important issues which need to be considered in mental health policy development [5]. The concept of a Mental Health Country Profile is similar to that of situation analysis in public health, where population needs and demands, existing services and current resources are assessed with the goal of change and improvement. This is particularly useful in countries which lack routine information collection, as the aim is to bring forth information and expertise which already exists in a country, but is often relatively inaccessible [5].

The aim of this study is to generate a systematic, integrated report of the mental health priorities in Vietnam, using the Mental Health Country Profile template.

## Methods

Vietnam has a population of 84 million inhabitants, and is the second most densely populated country in South-East Asia. Seventy three percent of the population lives in rural areas and the population growth is 1.21% per annum. The average income per capita is 750 USD, the maternal mortality rate is 130/100 000 live births, the gross domestic product is 638 USD per capita [6] and the poverty rate was 16% in 2006 [7]. Vietnam is a low income group country based on World Bank 2004 criteria, but due to extensive reforms in the past two decades, is on its way to becoming a middle income country [7]. Poverty remains much higher among ethnic minorities than among the Kinh and Chinese majority; constituting 14% of the population, ethnic minorities constitute 44% of the poor [7]. The country's capital is Hanoi in the north, and the largest city is Ho Chi Minh City (HCMC) in the south and the pace of rural-urban migration is rapid [7]. Lower fertility and improvements in healthcare are increasing life expectancy, and the resulting epidemiological transition from infectious diseases to non-communicable diseases will require a fundamental transformation in healthcare [7].

This study used snowball sampling [4,8] to identify informants, and data gathering was performed through semi-structured interviews and collection of reports and documents. We used the mental health policy template (table 1) as a basis for questioning. The interview questions were shaped along the template domains and elements, where first open-ended questions were posed, and emerging issues were clarified with follow-up questions. For example for the financial element of the resources domain, the open-ended question was posed: "what are the financial resources for mental health in Vietnam?". After this, follow-up questions could be posed, such as "are there any financial resources from non-governmental sources?" (for detailed interview guide see Additional file 1).

**Table 1:** Mental health policy template, adapted from Townsend et al. [11]

*Domains*	*Elements*
**Context**	*Societal organization and culture*
	*Public policy*
	*Governance*
	*Population need and demand*
**Resources**	*Financing*
	*Human resources*
	*Physical capital*
	*Consumables*
	*Social capital*
**Provision**	*Personal mental health services*
	*Population-based mental health services*
	*Intersectoral linkages*
**Outcomes**	*Health outcomes*
	*Service outcomes*
	*Economic outcomes*
	*Social outcomes*

Initially interviews were conducted by the first author with the Ministry of Health (MoH), World Health Organization (WHO), the National Institute of Mental Health (NIMH) representatives, and the director of the Traditional Medicine Institute. These sectors were deemed relevant primary actors in mental health, as they were the main sectors consulted in the original development of the Mental Health Country Profile methodology [5]. At the end of these interviews, informants were asked to recommend further informants.

When additional informants were recommended, they were immediately contacted for interview by the first author or by a research assistant from the Hanoi Medical University. All those who were approached agreed on participation in the study, and we aimed to interview all informants who had been recommended to us through the snowball sampling method (see table 2). There were two informants recommended however, who we did not succeed in scheduling an interview with due to time constraints; the head of the Institute of Psychology, and the mental health representative from the MoH Department of Therapy. Seven interviews were conducted in English and three were conducted in Vietnamese. Written consent was obtained from all interviewees beforehand and the length of the interviews varied between 45 minutes and two hours. At the English language interviews notes were taken, and these were transcribed and clarified later the same day. The Vietnamese language interviews were tape recorded, transcribed verbatim, and translated to English. All interviews were conducted at the informants' work places. When documents were mentioned by the interviewees, copies of these were requested. Thus any officially published documents or presentation overheads that were mentioned by the interviewees as relevant to the research question were included in the analysis. These documents formed additional material which complemented and detailed what had been said. Two informants were later asked to contribute to this paper as co-authors, and were chosen mainly because of their knowledge of scientific methodology (both have a PhD), and because their expert and mutually complementary views were deemed necessary to ensure the accuracy and validity of the findings. These co-authors reviewed the findings, and added up-to date information to the manuscript from their own fields of expertise. Ethical committees in Stockholm, Sweden and at the Hanoi Medical University, Vietnam approved the study.

**Table 2:** Interviews and manuscript revisions conducted

Institution/organisation*	Date
National Institute of Mental Health (NIMH)	Interview 09. 05. 2007

International Organisation of Migration (IOM, inter-governmental organization)	Interview 24. 05. 2007

World Health Organisation (WHO)	Interviews 24. 05. 2007 and 26. 02. 2008

Ministry of Health, Non-communicable disease program (MoH)	Interview 18. 05. 2007

Therapy centre, Hanoi (private initiative)	Interview 28. 05. 2008

Atlantic Philanthropies (non-governmental organization)	Interview 24. 05. 2007

National central mental hospital	Interview 04. 06. 2007

Traditional Medicine Institute	Interview 04. 06. 2007

Research and Training Centre for Community Development (RTCCD, non-governmental organization)	Interview 05. 06. 2007

Ministry of Labour, Invalid, and Social Affairs (MOLISA)	Interview 28.09.2009

Ministry of Health, Non-communicable disease program (MoH)	Manuscript revision 07. 10. 2009

Research and Training Centre for Community Development (RTCCD, non-governmental organization)	Manuscript revision 02. 01. 2010

*) Interviews were generally conducted with leading representatives of the institutions/organisations.

The collected material was analysed according to qualitative content analysis [4,9], where the interview transcripts and collected documents were first read through several times to attain a picture of the whole, and later, meaning units in the material were identified. Subsequently the meaning units were condensed into codes and divided into categories in accordance with the elements of the mental health policy template. As a check for validity of the results, the article manuscript was sent to all English-speaking informants for feedback before final revisions. However, only one informant in addition to the two co-authors provided comments, despite three e-mail reminders.

## Results

In the section below, the results of the data gathering are organised according to the domains and elements of the mental health policy template (table 1). When information has been taken from a document, its number is indicated in brackets according to those listed in table 3. Information from interviews is indicated with "(i)", but the interview which it comes from is not specified in order to ensure interviewee anonymity.

**Table 3:** Official documents collected for the mental health country profile

Document	Publisher
1. UN Youth Theme group, activities map 2006-2007	IOM

2. Ly Ngoc Kinh, and Vuong Anh Duong (2005) WHO-AIMS findings	WHO

3. T. V. Cuong (2002) Morbidity rate of mental diseases - results from a survey of 67,380 people in 8 geographical areas	Central Mental Hospital

4. Information sheet	IOM

5. Harpham T, Tuan T (2006) From research evidence to policy: mental health care in Viet Nam.	Bulletin of the World Health Organisation, vol.84 (8), pp: 1-5

6. L. D. Truong, WHO support for mental health care of Vietnam.	WHO

7. (2007) Diagnosis, treatment, care and management guidelines for patients with mental disorders in the community	MoH Community-based mental health project in collaboration with WHO.

8. Summary report of meetings, field visits and workshop on Community-based mental health care held in Viet Nam on 25-26 June 2006	IOM

9. T. V. Cuong (2004) report of the prevention and treatment of epilepsy and depression program.	Central Mental Hospital

10. Information leaflet	Department of psychology and education

11. (2007) Project: establishing the national integrated non-communicable diseases surveillance system (ndss) in Vietnam	MoH, prevention and control of non-communicable diseases program

12. H. Ngoc (2004) wandering through Vietnamese culture	The Gioi Publishers

13. T Tuan, L T Buoi, N T Trang. Evaluation of the community mental health project Cost-Benefit Analysis of Community-Based Mental Health Care Model A report to WHO Hanoi, March 2008	RTCCD

14. Mental Health Atlas 2005	WHO

15. Vietnam Development Report 2008: Joint Donor Report to the Consultative Group Meeting	Hanoi: World bank

16. T Tuan, J Fisher, Meena Cabral de Mello et al. (2005) Report of Workshop on Primary mental health care for mothers and children in Vietnam. Hanoi, June 6 - 10 , 2005	RTCCD

17. Harry Minas (2009). Reform of the MOLISA Centres for persons with severe mental disorders. Mission report to WHO & MOLISA, November 2009	MOLISA - WHO Hanoi

### Context

#### Societal organization and culture

The Confucian roots of Vietnamese culture have traditionally resulted in a sense of community (12). Traditional multi-generational households are dominant in the rural regions of Vietnam, but are becoming less so with increased urban migration, which also has consequences including increased unemployment and loss of social networks (15). Unlike for many other countries, the fraction of the economically active in Vietnam is the same for women as for men, but women are more likely to be in the lowest wage group (15). At the turn of the millennium, half of the Vietnamese population was under 25 years of age, but with declining fertility and increased longevity, the Vietnamese population is ageing rapidly (15).

#### Public policy

Vietnam's mental health policy was last revised in 1989 (2), and until 2004 it was a national plan of action on treatment of schizophrenia and epilepsy in hospitals. There were no health promotion or illness prevention strategies, and no community-based or primary care policies addressing mental health (5). The government has developed a 5-year national plan of action for 2006-2010, which incorporates mental health issues, and proposes to screen pregnant women and children for mental illness (5). Since 1945, guidelines to develop traditional medicine through research, promotion and integration with modern medicine have been implemented, and health insurance fully covers traditional medicine treatment and products (i).

There are no specific legal rights for the mentally ill, but the NIMH together with the MoH department of policy are in the process of devising them (6, 8). There are no alcohol policies, nor any policies to restrict discrimination (i). A national mental health human rights review body does not exist, but there is legislation to protect the human rights of patients. All hospitals have at least one review/inspection of human rights protection of patients per year (2).

A Community Based Mental Health Care Project (the National Target Program on Mental Health) was approved by the Government in 1999. It was initiated in 2000 for schizophrenia, and for epilepsy in 2004 (6, 8). The main objective of the program is to provide mental health services at the community level through mobilising community resources. Overall objectives until 2010 are to cover all communes and include epilepsy and depression in the project, though the focal point for the period 2006-2010 is schizophrenia (8). By June 2006, 3323 communes were covered by this project (13) and in 2009, the management model for epilepsy and depression had been implemented in 53 communes (i). The activities of this model include mental health training of health staff and health collaborators, as well as household surveys to identify depression and epilepsy patients, monthly delivery of medicines for patients, and monitoring and supporting patients through medicine and health education through village media.

#### Governance

(Here we refer to all bodies that act as to govern/exercise an influence over the national mental health services)

##### 1. Structure of Health System in Vietnam

The department of Medical Services Administration within MoH is the highest body of the health care system, and has main responsibility for developing policies relating to mental health, and monitoring and coordinating all activities. The department has a mental health advisory board, of which most members come from the national mental hospitals and the NIMH (8). The MoH also has a department of traditional medicine (i). The health care system is organized into four levels, the central, provincial, district and community level, and psychiatrists work at the central and provincial levels (i). The national psychiatric hospital controls the mental health system, manages other hospitals and conducts the national mental health program (8). More mental health services in Vietnam are provided in hospitals than in the community, but follow-up usually occurs at the community general practice (i).

The key actors in determining mental health policy in Vietnam are the following. The national assembly approves and monitors policy, and the communist party's central commission for science and education directs the development of health policy. The health strategy and policy institute at the MoH provides evidence base for policy formulation.

Apart from the MoH, other key players in mental health are as follows: The national committee for population, families and children deals with all sectors that have an impact on families and children (5). The Institute of Psychology does not belong to a ministry, but to the government directly, under the Vietnam Academy of Social Sciences (i). The Department of Social Affairs within the Ministry of Labour, Invalid, and Social Affairs (MOLISA) is responsible for mental health care rehabilitation through a national network of social protection centres for people with severe and chronic mental disorder, and a national program for people with severe mental illness providing support in the community (18).

##### 2. Non-communicable Disease Programme

A Non-Communicable Disease (NCD) program has been initiated in 2002, under the control of the department of Medical Service Administration. During the period 2002-2010, the NCD program focuses on hypertension, diabetes, some common forms of cancer and mental health (i). These four National Target Programs have so far only been implemented within the MoH and have not yet collaborated closely with other Ministries. An integrated model on NCD control was developed in 2006 and piloted in the community from 2007. By the end of 2008, mental health care was not included in the pilot project (14). The national surveillance system on NCDs was developed and initiated in 2007 and a pilot was implemented in 8 different geographical provinces (12).

##### 3. Nongovernmental players

National and international Non-Governmental Organizations (NGOs), multilateral organizations, and international universities have provided regular support for mental health issues in Vietnam (5). A national NGO, the Research and Training Centre for Community Development has had long term engagement with the government, resulting in the prevalence of statistics on mental illness among mothers and children being cited in the national plan of action (5). The International Organization of Migration is involved in capacity building and training of social workers and counsellors, and in child mental health work (1). The Atlantic Philanthropies is an international NGO, which has since 2006 funded the development of a mental health surveillance system (i). The WHO supports technical expertise and has initiated the development of the mental health law and of the NCD control model. The WHO is considering supporting MOLISA to reform the MOLISA centres for persons with severe mental disorders through a WHO-led rapid assessment (18). Today NGOs in Vietnam are suffering from decreased levels of international aid mainly due to the country's transition from being listed as a low-income country to a middle-income country (i).

#### Population need and demand

According to MoH statistics, mental hospitals in Vietnam hold three diagnostic groups of patients: schizophrenia, schizotypal and delusional disorders (60%), mood disorders (15%) and neurotic, stress-related and somatoform disorders (15%) (2). A clinical epidemiology investigation of common mental illnesses in 8 ecological regions was conducted in 1999 - 2001 (table 4) (9). There is little published scientific evidence about the extent and nature of mental health problems in Vietnam (5).

**Table 4:** Results of clinical epidemiological survey on some common mental disorders (9)

*Illness*	Rate, %
Schizophrenia	0,47
Epilepsy	0,35
Memory-loss by age	0,88
Depression	2,8
Anxiety	2,6
Behaviour disorder in youth and teenager	0,9
Alcohol abuse	5,3
Drug addiction	0,3

**Total**	13,6

### Resources

#### Financing

In the past, medical care in Vietnam was free at all levels. However, after the adoption of the economic renovation policy in 1986, only a part of patients' medical costs have been shouldered by hospitals, while private for profit clinics have been permitted to open. Mental hospitals are entirely subsidised by the government (i). The government only pays for control and medication of epilepsy and schizophrenia, while medication and treatment for other mental illnesses is paid out-of-pocket (9). However, within the MoH National Target Program on mental health, psychiatric medicines were provided free of charge.

The government spends approximately two million USD per year on mental health (compared to 46 million USD allocated in Thailand and no allocations in Laos in 2004 [3]), while some additional financing comes from international donors (i). For example, the WHO had by 2007 funded mental health in Vietnam with a total of 80 000 USD (i).

#### Human resources

In Vietnam there are both psychiatrists, who specialise for three years, and psychiatric doctors who receive one year of training in psychiatry (i). General practitioners (GPs) receive one month of mental health training. Thus they learn to use WHO mental health surveys and the ICD-10 version for community health care. In addition, training is organised yearly for practicing GPs to increase their knowledge about psychiatry (i), though this training focuses solely on psychotic disorders (13). In the training programme for nurses, one percent is devoted to psychiatry (2). In addition, there is a Master's and PhD programme in psychiatry, coordinated in collaboration between the Ministry of Education and Training and the MoH.

In 2004, there were 0.32 psychiatrists per 100 000 population (compared to 11 in the UK and 13.7 in USA [3]), 0.03 neurosurgeons (compared to 1 in UK and 1.6 in USA (ibid.)), 0.3 psychiatric nurses (compared to 104 and 6.5 (ibid.)), 0.13 neurologists (compared to 1 and 4.5 (ibid.)) (14). Other human resources for mental health include 0.03 psychologists per 100 000 population (compared to 9 in the UK and 31.1 in USA (ibid.)), 125 social workers (compared to 58 per 100 000 population in the UK and 35.3 in USA (ibid.)) (14), 4 occupational therapists (2), and traditional medical practitioners (i). Psychologists learn general psychology but not clinical psychology and social workers rarely work with mental health (i). MOLISA has recently focused on the development of social work in Vietnam, as marked by a national workshop organized in October 2009 in Da Nang (18). The Community Based Mental Health Project involves the training of local staff in mental health, including health workers, social workers and members of the women's unions (i). Individual efforts have resulted in therapy and counselling services opening around the country, such as the individual family and counselling clinic (IFC) in Ho Chi Minh City since 2002 (i), the TuNa Clinic specialising in mental health care for mothers and children in Hanoi since 2005 (i), and a therapy centre in Hanoi since 2006 (i).

#### Physical capital

There are 64 provinces in Vietnam and in 27 of these, there is a separate psychiatric hospital, while in the rest of the provinces psychiatric care occurs at the district hospital (i). Together these hold 0.63 beds per 10 000 population (compared to 5.8 in the UK and 7.7 in USA) (14). There are three hospitals that operate under the MoH, and these include the two central mental hospitals in Ha Tay in the north, Bien Hoa in the south and the NIMH at the Bach Mai hospital, Hanoi (8). There are three independent mental health dispensaries in three provinces, 10 mental health dispensaries in 27 provincial mental hospitals, and 25 mental health departments in social diseases prevention centres. Two provinces do not have a mental hospital or department (8). There are 600 outpatient mental health facilities and two day treatment facilities and 20 community-based psychiatric inpatient units with a total of 300 beds. There are 300 beds for persons with mental disorders at forensic inpatient units (2). There are no private psychiatric hospitals in the country (i)

Psychiatric practitioners are trained at three medical universities in Vietnam; HCMC Medical University, Hanoi Medical University, and Hue Medical University (i). Psychologists are trained at the department of psychology and education (Hanoi) (10) and the University of Pedagogy and Psychology (HCMC) (i). There are approximately 30 social work programs now in the whole of Vietnam (i). There is a department of traditional medicine at Hanoi Medical University, and every hospital in the country has a traditional medicine department. In addition, each province has its own traditional medicine department (i). There are also public short training courses on primary mental health care launched by the NGO Research and Training Centre for Community Development since 2006.

#### Consumables

The supply and pricing of psychiatric medicines is regulated by the Vietnamese government, and the cost of antipsychotic medication is 33% of 1 day's minimum wage. All mental health outpatient facilities have psychotropic medicine, including anti-psychotic, antidepressant, mood stabilizer, anxiolytic and antiepileptic medicines. Mental hospitals generally hold enough psychotropic medicines, and 51-80% of primary health care facilities have at least one psychotropic medicine of each therapeutic category (2). The national target is to reach full health insurance coverage by 2010, but by 2009 only close to half of the population is covered.

In 2007 the MoH has together with the WHO authored a document with basic guidelines for the treatment, care and management of mental disorders in the community (7). This document is primarily based on the ICD-10, DSM-III-R and DSM-IV-R, and is to be distributed to GPs at the provincial and district levels (7). In the field of psychotherapy, there is a marked lack of textbooks and journals translated into the Vietnamese language (i). The NGO Research and Training Centre for Community Development has translated two books to Vietnamese; the WHO "Primary Care for Mental Disorders" was translated in 2006 and is used as the key book for training health staff and social workers on primary mental health care. In 2009 "Where there is no psychiatrist: A Mental Health Care Manual" (Patel 2003) was translated and will be distributed to community health workers by the NGO Vietnam Veterans American Foundation (i).

#### Social capital

(Here we refer to any resources that are available for mental health management in terms of mutually advantageous connections between individuals or within social networks)

No official consumer or family associations exist for the mentally ill (2), though recently, a group of families with autistic children in Hanoi started to work together, exemplifying a trend of establishing civil organizations on mental health care (i).

### Provision

#### Personal mental health services

(Here we refer to the mental health initiatives that are aimed at the individual at a personal level)

In 2004, 54 500 patients were treated in mental hospitals, and the average number of inpatient days was 35. There were 46 070 patients treated within outpatient facilities, and at day treatment facilities, there were 3.7 users per 100 000 population (2). One percent of admissions to mental hospitals are involuntary, and two to five percent of inmates are restrained or secluded (2).

The medical management of mental illness in Vietnam only involves medication, and there is no family education or psychotherapy (i). Only doctors are allowed to prescribe psychotropic medication. Those psychologists who work in hospitals are engaged in clinical testing. About 5% of patients in community-based inpatient units, and 60-70% of patients at mental hospitals received one or more psychosocial intervention in 2004 (2).

Twenty percent of the physician-based primary health care services include complementary/alternative/traditional practitioners (2). Traditional medicine is used for neurasthenia, and dissociative disorders and treatment consists mainly of acupuncture, massage and herbal medicines. Patients with schizophrenia, personality disorders, paranoia, or suicidal thoughts are not treated by traditional medicine (i).

#### Population-based mental health services

(Here we refer to the mental health initiatives that are aimed at the population as a whole, or specific groups within the population)

There are no regular national programmes for information on or promotion of mental health, though public education and awareness campaigns have been held targeting the general population and health care providers, leaders and politicians (2). Public programmes, including information panels, booklets given out to patients and their family, and talks on national TV and radio have been organised by the central mental hospital (9). However, this has mainly focused on psychotic disorders within the context of the Community Based Mental Health Project (i).

#### Intersectoral linkages

The mental health sector collaborates formally with sectors responsible for primary health care/community health, reproductive health, child and adolescent health, substance abuse, child protection, employment, welfare, criminal justice and the elderly. Mental health providers interact with primary care staff (2). United Nations organizations, the WHO and NGOs are collaborating with the central mental hospital and NIMH on the assessment of the Community Based Mental Health Project (i). However, there is weak collaboration between MoH and MOLISA for care of severe cases of mental disorders after discharge (i). The engagement between researchers and policy-makers has been initiated, and networks of key stakeholders have been established (5).

### Outcomes

#### Service outcomes

An evaluation of the Community Based Mental Health Project by the Research and Training Centre for Community Development has shown that the model has only been implemented for schizophrenia, and training has been provided only for health care personnel, and not other community groups. Moderate and severe cases of schizophrenia are being relatively well managed through the model, but epilepsy is under managed and monitoring and supervision of the activities is poor. The model has had little impact of increasing the numbers of suspected schizophrenia and epilepsy cases being referred to professional care and diagnosis. However, medicines in communes where the project has been implemented are distributed at a commune level, while otherwise drugs are obtained from the provincial level. Duration of inpatient care is shorter and severity of illness has been decreased in the implementing communes (14).

The WHO has encouraged the Vietnam government to develop mental health care in all hospitals instead of separate asylum-type tertiary hospitals to decrease the isolation and stigma attached to the mentally ill (i). Additional WHO recommendations include providing treatment at the primary care level, increasing the availability of psychotropic medicines, developing community-based care, training, recruiting and providing sufficient pay for professionals, and educating the general public on mental disorders (7). Through collaboration between researchers and policy makers, the main gaps identified are the lack of knowledge about the feasibility and cost of any intervention (5).

## Discussion

### Methodological considerations

The quality of any policy analysis depends on the accuracy, comprehensiveness and relevance of the information collected [4]. Information on developing countries' mental health system is often patchy, disorganised, inaccurate and not triangulated or discussed with key stakeholders [5]. This study aimed to tackle these common shortcomings through triangulation of sources [10]. We find that we have obtained a relatively detailed and nuanced view of the mental health situation through our snowball sampling method. Nevertheless, it is unlikely that all relevant views have been heard and there were for example two informants who we did not succeed in interviewing.

A weakness of this study is that it was conducted solely in the Hanoi area. Though Hanoi is the capital of Vietnam, and most governmental and non-governmental institutions have their head office there, we may have gained a broader perspective by interviewing representatives from the HCMC area as well. Interviews within the health care system were only conducted at the central and governmental levels. Additional perspectives may have shed more light on the process of implementation within the mental health care system. Figures concerning budgetary allocations for mental health can be seen only as rough estimates, as they were based on verbal accounts alone.

There were some challenges involved with using the mental health policy template as a framework for the content analysis, mainly due to that the domains and elements of the template described in the literature are generic and not specific for the Vietnam setting [11]. We tackled these challenges through an inductive process whereby already published Mental Health Country Profiles that have used the template were read thoroughly in order to understand the ways in which the domain and element contents can vary between contexts. In order to clarify the meaning of the different domains and elements, we have made an effort to explicitly describe those element titles of the template that we did not deem self-explanatory. Due to its generic nature, we deem that the template is in many ways useful for the logical structuring of the different components of a Mental Health Country Profile.

We gave all interview participants an opportunity to give feedback on the final manuscript, apart from two participants who did not speak English, as this would have increased the validity of the study. However, only three informants provided feedback despite three reminders and the reasons for this remain obscure

In the following section we discuss the results with special regard to the adequacy of the policies and provision of mental health services in Vietnam, mainly in light of international recommendations and findings from research in cross-cultural contexts.

### Context

According to the WHO [12], reducing stigma should be globally prioritised as a population-based approach to improving mental health. In Asia, the tendency to stigmatise and discriminate against the mentally ill is prevalent, and concerns not only the mentally ill, but also their families, and the pathway to care is often impeded by scepticism towards mental health services and treatments [13]. Mass information campaigns may be good for tackling stigma, as ignorance, cultural stereotypes and myths lead to prejudice [14]. Thus public information programmes are recommended but have not yet been realised in Vietnam. Importantly, the transition from large tertiary psychiatric hospitals would also help to reduce stigma.

### Resources

To fully describe mental health system performance, it is important to acknowledge the role of the private sector, civil society and the local community [15]. In this study we found that NGOs and multilaterals play an important role in enhancing mental health care in Vietnam, especially in the community and primary care settings, through financing, goal-setting and provision of care such as psychotherapy.

The Community Based Mental Health Project initiative indicates an evolvement from large psychiatric hospitals towards care in the community, in line with WHO recommendations [12], though no efforts were found of closing of large tertiary hospitals. Additional steps towards community based management are the relatively good availability of psychotropic medication at primary care, increased training of general practitioners in mental health care, and the development of diagnosis and treatment guidelines for community care. In Vietnam, most essential medications can be found in most parts of the country [16] and it is mainly the high price of psychotropic medications that poses a problem since for many conditions they are paid out-of-pocket.

### Provision

According to Jenkins et al. [5] for the public health burden of mental illness to be tackled effectively, governments should engage in much more than just curative services for the acutely ill. The Vietnamese government has not come very far in this task, though efforts are being made through the Community Based Mental Health Project. Within the governmental sector, it seems that the concept of mental disorders prevention has not been realised. There is no link between the MoH mental health care services and other sectors on this aspect, and no documents are available for the public on the prevention of mental disorders. Alcohol abuse is a relatively large burden in Vietnam, as illustrated by the 5,3% prevalence rate. Policies to reduce alcohol abuse, such as restrictions and increased taxation could be effective in reducing this burden [17].

### Outcomes

The Community Based Mental Health Project implementation has not yet focused on depression, though it is the leading cause of disability across the world [18], is strongly associated with poverty [12], and primary care interventions for depression can be as cost-effective as anti-retrovirals for HIV/AIDS [17]. The lack of focus on depression treatment mirrors the situation found in most low-income countries, where this illness receives little programmatic and research attention [19]. Though there is limited research evidence of cost-effective depression treatment - which was also found in the present study - a number of studies have shown that combining medication with locally feasible psychological interventions can be effective and cost-effective among the poorest people in a low-income country, and produce significant reductions in total health care costs [17,19].

The Millennium Development Goal #5 is the least developed of all eight and the rates of reduction of maternal mortality are too slow to meet the goals of reducing the ratio by three quarters by 2015 [20]. About 1 in 4 mothers suffers from depressive disorders at some point during motherhood, and even more so in low-income countries [21]. Thus, it is positive that advocacy from the RTCCD has lead to statistics on mental illness among mothers and children being cited in the national plan of action.

## Conclusions

We have aimed to highlight some strengths and weaknesses contributing to a Mental Health Country Profile for Vietnam to assist future efforts and decision-making. This initial attempt to map the mental health situation of Vietnam suffers from a number of limitations and should be seen as a first step towards a comprehensive profile.

Marked strengths of the Vietnam mental health system are the aims to move toward community management and detection of mental illness, and the active involvement of several multilateral organizations and NGOs. Nevertheless, mental health care in Vietnam is still characterised by unclear policy and poor critical mass especially within the governmental sector. Drawing from the findings of the present study, we would like to make the following recommendations for improving the mental health system in Vietnam: 1) development and provision of locally feasible and effective non-pharmaceutical treatment interventions; 2) increased health insurance coverage of treatments, including pharmaceuticals for common mental disorders; 3) replacement of care in large tertiary hospitals with other, less stigmatising forms of service provision; and 4) increased commitment in preventative measures for mental illness including increased mental health information provision to the general public.

## Competing interests

The author Tran Tuan is director of the Non-governmental organization Research and Training Centre for Community Development and the author Tran Huong is coordinator of the Non-communicable Disease Programme at the Vietnam Ministry of Health. Other authors have not declared any conflicts of interest.

## Authors' contributions

MN designed the study, conducted the interviews, performed the data analysis and drafted the manuscript. HT and TT both contributed to data analysis and interpretation, and contributed to drafting and revising the manuscript. TT conducted one interview. TF conceived of the study, participated in design and coordination and helped to draft the manuscript. All authors read and approved the final manuscript.

## Pre-publication history

The pre-publication history for this paper can be accessed here:

http://www.biomedcentral.com/1472-6963/10/257/prepub

## Supplementary Material

Additional file 1 **Interview guide for data collection**. Click here for file
