# Supplementary material for: Mental health priorities in Vietnam: a mixed-methods analysis
Source: BMC Health Serv Res. 2010 Sep 2;10:257. doi: 10.1186/1472-6963-10-257 (PMC2942883; doi:10.1186/1472-6963-10-257)
Supplement: Additional file 1 — Interview guide for data collection. [file 1472-6963-10-257-S1.DOC]

**Interview guide for data collection**

The following interview guide was followed for all interviews. However, for each individual interview, only those questions that were deemed relevant were selected from the guide. Follow-up questions were posed whenever relevant or interesting issues emerged.

| **Area of interest** | **questions** | **probes** |
| --- | --- | --- |
| **Context – societal organization and culture** | What are the prevalent Vietnamese attitudes toward mental health? | - Is mental health seen as important? - Why, what are the main factors influencing these attitudes? |
|  | What are the main value systems that influence views on mental health in Vietnam? | - Is mental health regarded as important? - More or less important than physical health? |
|  | What are the predominant cultural views in Vietnam about the causation of mental illness? |  |
|  | What are the social issues in Vietnam that cause mental illness? | - For example: alcohol or drug abuse, issues of injustice, etc? |
|  | What are the prevalent religious views on mental health in Vietnam? |  |
|  | Are the mentally ill discriminated against in society? | - In education? - In work life? - In other sectors? |
| **Context – public policy** | Is there a national mental health policy? | - Who has devised this policy? - What are the priorities listed in this policy? |
|  | What are your organization’s priorities regarding mental health in Vietnam? | - How much money does your organisation spend on mental health in Vietnam per year? |
|  | What kind of policies are there in Vietnam that have an impact on mental health? | - Drug and alcohol policies? - Policies to restrict discrimination? |
|  | Are there any specific mental health programmes in Vietnam? |  |
|  | What are the rights of the mentally ill, e.g. to confidentiality etc? | - What regulations are these rights based on? |
| **Context - governance** | How is the Ministry of health in Vietnam organised? | - What are the bodies that regulate its practice, and what are the bodies that act under it, e.g. at the provincial and district level? |
|  | What are the political factors and levels of government that influence mental health services in Vietnam? |  |
|  | What are the main institutions that influence mental health services in Vietnam? | - Governmental organisations? - NGOs? |
| **Context - Population need and demand** | Is there data available of the mortality, morbidity, and service use of the mentally ill? Is there knowledge about the prevalence of mental disorders? | - Could you provide this data? |
|  | Have there been national surveys or research conducted on the prevalence of mental illness in the country? | - What research/surveys? - What were the results? |
| **Resources - financing** | How many percent of the GDP is spent on health? And how many percent of this is spent on mental health? |  |
|  | How is mental health care funded? |  |
|  | How is traditional medicine funded? |  |
| **Resources – human resources** | How many trained mental health professionals are there in Vietnam (psychiatrists, neurologists, psychologists etc.)? | - Where are these professionals trained? - How are the mental health staff geographically distributed in Vietnam? - At what levels of the health service is mental health care provided? |
|  | To what extent are family doctors and general practitioners trained in mental illness diagnosis and treatment? | - What does the training consist of? |
|  | At what levels of health care do psychiatrists work? |  |
|  | How many traditional medicine doctors are there in the country? | - Where are traditional medicine doctors trained? - How long is the training? - Where is traditional medical care provided? - Is traditional medicine provided for the treatment of mental ailments? If so, which ones? |
|  | How many social workers and councillors are there in Vietnam? | - Where are they trained? - What do they usually work with? |
|  | How is the quality of mental health care regulated in Vietnam? | - How is the practice of mental health professionals regulated? |
| **Resources – physical capital** | How many psychiatric hospitals are there in the country? How many out-patient dispensaries? How many psychiatric beds are there for in-patients? |  |
|  | Are diagnostic tools such as EEG and ECT readily available? | - Where? |
| **Resources - consumables** | How available are medications? | - Who is responsible for the supply, pricing and dispensing and registration of medications? - What do psychiatric medicines cost, and are they paid out-of-pocket? |
|  | Are text books of psychiatry and psychology, and diagnostic guidelines of mental illnesses readily available in Vietnamese? |  |
| **Resources – social capital** | What are the main support systems that exist for the mentally ill? | - unofficial and official? |
| **Provision – personal mental health services** | What kind of health care do the mentally ill seek? |  |
|  | What kind of other care do the mentally ill seek? | - Traditional medicine? - Spiritual or religious services? - Why do people seek care from these providers? |
| **Provision – population-based mental health services** | At what levels of the health care system is mental health care provided? |  |
|  | How many people are covered by each community health centre, and district level hospital? |  |
|  | How do patients come to seek mental health care? | - Are they referred from a doctor? - Do they refer themselves? - Is there involuntary admission, and if so, how does it work? - What kind of follow-up and rehabilitation is there for psychiatric patients? |
| **Provision - Intersectoral linkages** | Are there any public information or mental health promotion programmes in Vietnam? |  |
|  | What other government ministries does the MoH interact with on mental health issues? |  |
|  | Which are the other ministries which work with issues of relevance to mental health? |  |
| **Outcomes** | Have the national mental health programmes been evaluated? | - What were the outcomes? |
|  | Have the NGO and private mental health initiatives been evaluated? | - What were the outcomes? |
| **Snowball sampling** | Could you recommend any other key players in mental health that I should interview, in order to obtain more information on these issues? | - Concerning the issue XX that we discussed? - Concerning the issue YY that we did not discuss? |
